# Interrelationships and Trade-Offs between Urban Natural Space Use and Biodiversity

**DOI:** 10.3390/su16104051

**Published:** 2024-05-02

**Authors:** Elena Prioreschi, Nici Zimmermann, Michael Davies, Irene Pluchinotta

**Affiliations:** Institute for Environmental Design and Engineering, The Bartlett Faculty of the Built Environment, University College London, London WC1H 0NN, UK

**Keywords:** urban natural space, biodiversity, systems thinking, unintended consequences, causal loop diagrams, natural space management

## Abstract

Urban natural spaces provide important ecosystem services and a wide range of health- and well-being-related benefits for their visitors. They are also essential spaces for biodiversity protection and promotion in a world of rising urbanisation rates and worsening impacts of climate change. However, these spaces are often underutilised by urban residents. When they are utilised, this usage often leads to some level of environmental degradation and biodiversity loss. Hence, understanding how to promote both use and biodiversity levels in urban natural spaces is critical. While various reports have studied the broad factors associated with urban natural space use, the specific relationship between biodiversity and use remains to be explored. This paper uses a Systems Thinking approach to unpack the complex relationship between urban natural space use and biodiversity and to help guide the design and management of these spaces in a way that promotes both use and biodiversity. With data collected from a systematic literature review, a causal loop diagram (CLD) was constructed and analysed. The CLD construction and analysis highlighted various key factors that play an important role in relating urban natural space use and biodiversity. Among these is the role of individual and social perceptions and values in determining how biodiversity levels will affect usage, and vice versa. The results were applied to a case study: the Thamesmead regeneration project undertaken by the social housing association Peabody. We made recommendations regarding Peabody’s biodiversity and green infrastructure plans for Thamesmead, presenting new design and maintenance techniques and assessing various existing techniques mentioned in the documents. Through the CLD analysis, we uncovered various unintended consequences from common design and maintenance techniques and discuss these trade-offs and relationships.

## 1 Introduction

With accelerating global urbanisation rates and loss of biodiverse habitats to urban development [1], interest in preserving wild areas in cities has increased in recent years [2]. Urban natural spaces are becoming essential places for biodiversity promotion in cities [3] and provide a wide array of benefits such as increasing resilience to climate change [4–6]. Further than biodiversity promotion, urban natural spaces provide nature contact for urban residents [6], helping improve human health and well-being [7].

Research has shown that cities can support a high level of biodiversity, providing essential habitat for many species, including threatened and endangered species [8,9]. Biodiversity encompasses the genetic variation found among all living things on Earth, from animals and insects to microbes, plants, and fungi and the ecosystems they comprise [10]. Biodiverse spaces provide ecosystem services necessary for life on Earth—providing regulating services (e.g., pollination, air purification, and climate regulation) and provisioning services (e.g., food, fresh water, and fuel).

The importance of human contact with natural spaces has been well established in the literature. For instance, contact with blue and green spaces has been shown to restore concentration [11], reduce stress levels [12], and improve cognitive function [13]. In addition to the direct physical and mental health benefits, nature contact offers many cultural ecosystem services that allow for social cohesion and connection among people [14].

Despite the wide array of benefits resulting from nature contact, urban natural spaces are often underutilised by urban residents [15]. Daily contact with nature is becoming increasingly rare, especially among children—constituting a phenomenon known as the “extinction of experience” [16]. Losing contact with nature is associated with many negative consequences: In addition to surrendering the aforementioned public health and well-being benefits, loss of nature contact lowers people’s emotional affinity toward nature, leading to less support for environmental movements and policies [17]. Evidently, finding ways to support and promote the use of urban natural spaces by urban residents is crucial in supporting both human and planetary health, if the two can even be considered separately.

For the purposes of this study, use of urban natural spaces is defined as any type of visit to an urban natural space, regardless of the purpose or length of the visit [18]. Utilisation of urban natural spaces is dependent on several factors, from physical aspects of the area (e.g., accessibility, quality of natural aspects, and amenities) to individual elements (e.g., individual values, life stage, and health) [19]. These factors are interlinked, forming a complex web that influences people’s usage of urban natural spaces. Throughout the literature, a knowledge gap has been repeatedly identified: the relationship between biodiversity and the use of urban natural spaces [19,20]. Though various studies have focused on topics relating a specific biodiversity-related factor with a use-related factor, a holistic system-level understanding is lacking [10]. For instance, the perceived level of biodiversity has been shown to directly influence the aesthetic quality of the spaces [21], the attractiveness of parks for both leisure [22] and physical activities [23], and the perceived value of these spaces [24]. Also, the recreational use of these spaces has been tied to various biodiversity-related impacts, such as vegetation loss [25] and eutrophication of water bodies [26]. However, how these individual relationships relate to each other and the wider system has yet to be explored.

Given the complexity of the interdependencies between biodiversity and usage of natural urban spaces, applying a Systems-Thinking modelling approach may enable a deeper understanding, providing clarity of the causality chains influencing the system under consideration. Systems Thinking is a long-practiced interdisciplinary method of understanding complex systems, generally with the goal to influence and design strategies and guide change [27]. Systems Thinking allows for factors within systems to be mapped and the effect of changes among these factors to be uncovered [28]. The impact of potential strategies can be investigated through Systems-Thinking modelling, with the aim to uncover any associated unintended consequences in the process [29].

Within Systems Thinking practice, it is common to build causal loop diagrams (CLDs). CLDs are maps that visualise the structure and causality between different components of the system of interest [27]. CLDs serve as a type of map, in this context providing a visual description of the complex set of variables and interconnections between urban natural space use and biodiversity.

CLDs and Systems Thinking have been used to support decision making and urban policy assessment surrounding climate change, environmental management, and nature-based solutions (e.g., [19,30–32]). In this work, constructing and analysing a CLD sheds light on factors that could be adjusted to improve the usage of urban natural spaces while also understanding the factors’ impacts on biodiversity.

This paper aims to apply Systems Thinking to systematically map the causal links and interdependencies between urban natural space use and biodiversity to (a) unpack the complexities of the relationship and (b) guide urban natural space design and maintenance practices to promote both use and biodiversity.

The paper firstly builds and analyses a CLD that relates urban natural space use and biodiversity by (a) determining the main variables at play in the relationship via a systematic literature review; (b) constructing a CLD that maps the causal links between the identified variables; and (c) analysing the results and investigating various links further, determining the relationships that may be most applicable in guiding urban natural space design and maintenance practices. Up until this point, the knowledge gathered to develop the CLD is generalisable. Next, the CLD results are applied to a case study: the Thamesmead regeneration project in London, U.K. We consider the impacts of the CLD findings on urban natural space use and biodiversity in the Thamesmead case study and provide recommendations considering two strategy documents published by the developer, Peabody [33,34]. This analysis assesses potential unintended consequences associated with the recommended design and maintenance techniques, providing an example application of the CLD results.

The paper is structured as follows. The study methodology is presented in Section 2, describing the data collection through a systematic literature review, open coding process to convert the data into a CLD, and finally, the methodology of CLD analysis. Section 3 provides (a) general insights from the causal loop analysis; (b) a description of major variables and feedback loops included in the CLD; (c) a closer look at three major feedback loops and their implications for urban natural space design and policy; and (d) application of the CLD results to the Thamesmead case study. Section 4 focuses on the role of individual and social values in biodiversity conservation and urban natural space use. The paper concludes with a summary of the study limitations and recommendations for future work.

## 2 Methods

**Figure 1:**
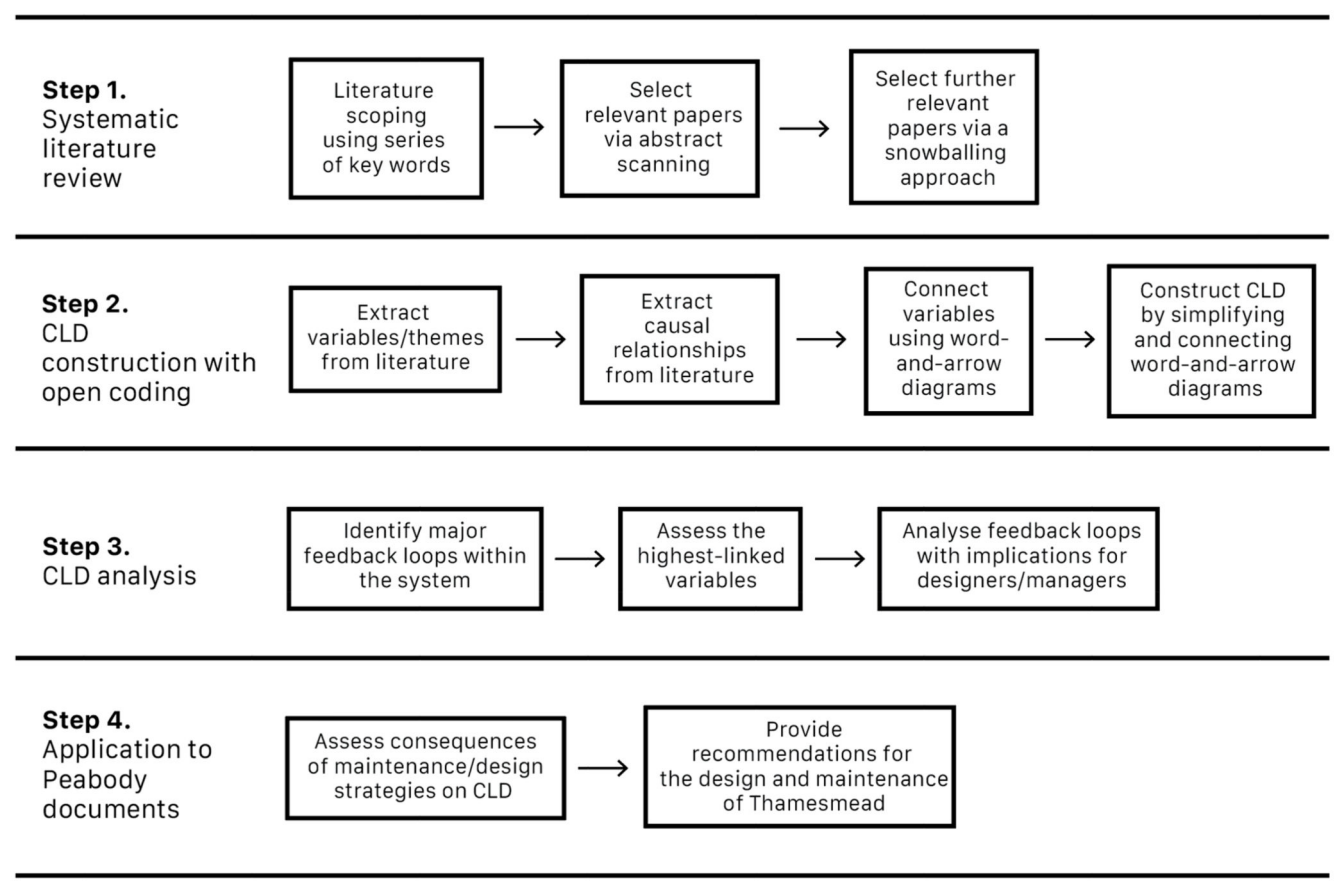
Study methodology workflow divided into four major steps: (1) Systematic literature review; (2) CLD construction; (3) CLD analysis; (4) application of results to Peabody development plan.

We applied Systems Thinking to unpack the complex relationships between factors affecting urban natural space use and biodiversity, building a CLD [27]. CLDs represent systems using three basic elements: nodes, connections, and feedback loops [35]. Nodes are relevant variables within the system, and connections are tied to polarities between the variables, depicting causal relationships [36]. CLDs provide valuable insight into complex systems, acting as a visual aid and map that display the known and perhaps lesser-known or even hidden interdependencies of variables within a system [37]. CLDs can be constructed from a wide range of data, from interviews or workshops with stakeholders to qualitative data drawn from the literature [38]. For this study, given the availability of relevant data in the literature, we collected data via a systematic literature review. The CLD was constructed following the methodology developed by Kim and Andersen [39], a study devising a method for creating CLDs from purposive text data. Once we constructed the CLD, it was analysed via a methodology similar to that described in Salvia et al. [19]. Figure 1 provides a depiction of the step-by-step methodology applied in this paper.

Since the methodology to build and analyse the CLD is replicable in other contexts, the following sub-sections provide an in-depth description of each modelling step.

### 2.1 Systematic Literature Review

**Figure 2:**
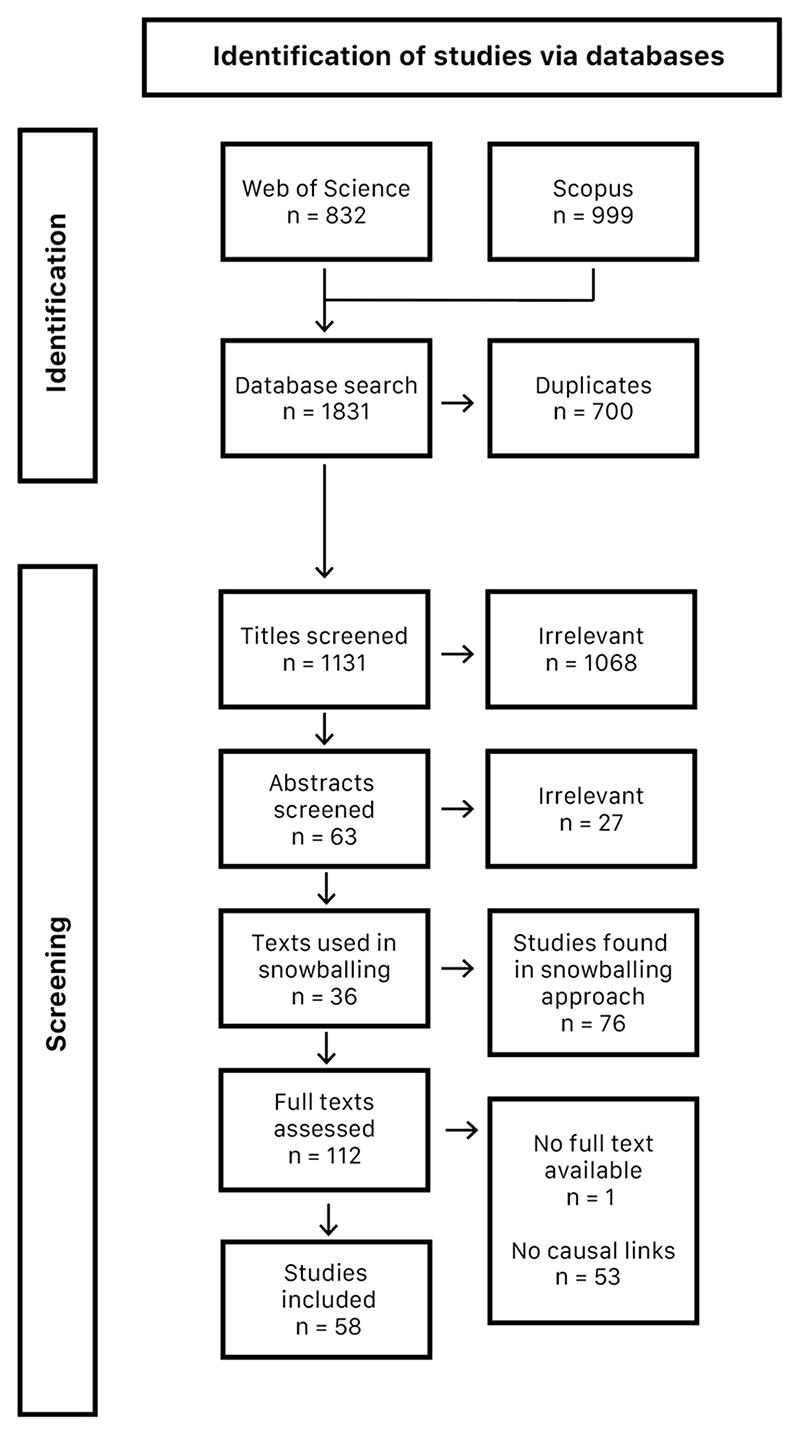
Prisma diagram detailing study selection process for systematic literature review.

The systematic literature review aimed to explore the current understanding of the relationship between urban natural space use and biodiversity as well as to identify major knowledge gaps. The primary literature search aimed to find peer-reviewed papers and reports involving information related to both biodiversity and urban natural space use. The primary search involved the key words detailed in Table 1, entered into databases Web of Science and Scopus. Figure 2 details the full literature search process.

From an initial group of 63 papers collected, we selected 36 papers for relevancy through abstract scanning. To further extend the search, we applied a snowball approach, and 76 further papers were selected as pertinent papers to the topic, creating a group of 112 papers. Of this group, 58 papers included relevant causal relationships, thus composing the final group of studies used to build the CLD. Though there were no formal quality criteria for inclusion or exclusion, as in most systematic literature reviews, we consider this a systematic literature review due to the extent and depth of the synthesis conducted during the construction of the CLD. The final group of 58 studies were all published between 1988 and 2022, with 80% published in the last decade. The inclusion of some older publications allowed the analysis to observe how perceptions have changed over time and how some ideologies still ring true many years later.

### 2.2 Building the CLD

With the final group of papers selected, the process of forming the CLD began, following a similar methodology of open coding as Kim and Andersen [39]. Kim and Andersen [39] developed a systematic method to generate causal maps out of qualitative data, with an open coding process that aims to reduce modeler bias when developing variables from text. The first step of the CLD construction involved an open coding process in which we identified variables from the literature. We created a coding table to record the various sources employed to identify these variables (Appendix A). To reduce coder bias, we carefully studied the context of statements within the literature to avoid incorrectly assuming implicit structures, as recommended by Kim and Andersen [39]. An implicit structure is the discovery of a variable that lies between two variables in a previously defined relationship. Uncovering implicit structures may allow variables to be linked where they were previously thought to be unrelated.

Once the pertinent variables were defined, we derived causal relationships from the literature (Appendix B). Defining causal relationships allowed the variables to be connected via cause-and-effect linkages. Each relationship has a positive or negative effect. A positive link signifies that if there is an increase in the cause variable, the effect variable will increase more than what it would have otherwise and similarly for decreases in the cause variable. Negative links indicate that if there is an increase in the cause variable, the effect variable will decrease more than it would have otherwise. By specifying that variables change “more than they would have otherwise”, we acknowledge that variables are influenced by other factors in the system and not only the link in question. Additionally, we acknowledge the limitations of CLDs in that they do not specify differences between stocks and flows [27].

With the causal relationships defined, we transformed the text into word-and-arrow diagrams. Word-and-arrow diagrams allow causal relationships between two variables to be visualised, connecting them with arrows and indicating whether their relationship is positive (with a “+”) or negative (with a “*−*”). In this step, overlap between relationships can occur, leading to the need for simplification and removal of any duplicate links in the CLD [39]. We constructed the CLD with the System Dynamics software Vensim, Version 9.1, “https://vensim.com/” (accessed on 3 July 2022).

### 2.3 Analysing the CLD

For the CLD analysis, we implemented both qualitative and quantitative methods. Following a similar methodology as Salvia et al. [19], the analysis included the identification of major feedback loops and an assessment of the highest-linked variables. We separated the variables into thematic clusters to identify trends or recurring patterns, using a process of individual clustering and group validation to minimise modelling biases.

We identified the highest-linked variables through the computation of a factor known as degree centrality (DC). DC is a simple calculation of the number of connections a variable has within the CLD (a summation of in- and out-arrows). Assessing the DC of variables provides valuable insight into the complexity of network links and which variables could affect many other variables in the system. While DC is an important indicator of connectivity, considering the number of loops a variable exists within can shed light on the overall reach a variable has within the system [40]. For this reason, the number of loops involving each variable was calculated, analysed, and discussed.

For the identification of feedback loops, we used the software Vensim. We further analysed the CLD for feedback loops that may provide insights for urban natural space designers, managers, and decision makers and, finally, with specific regard to the Peabody documents. Feedback loops can be in the form of reinforcing or balancing loops. Reinforcing loops are positive feedback loops in which the variables at play in the loop reinforce one another, leading to amplifying effects in the process until a constraining or balancing factor is introduced (external to the loop). Balancing loops include variables that limit or balance the growth of the variables within the loop as well as applying growth-stopping pressures on the rest of the system [41].

### 2.4 Application of CLD Results to Thamesmead Case Study

**Figure 3:**
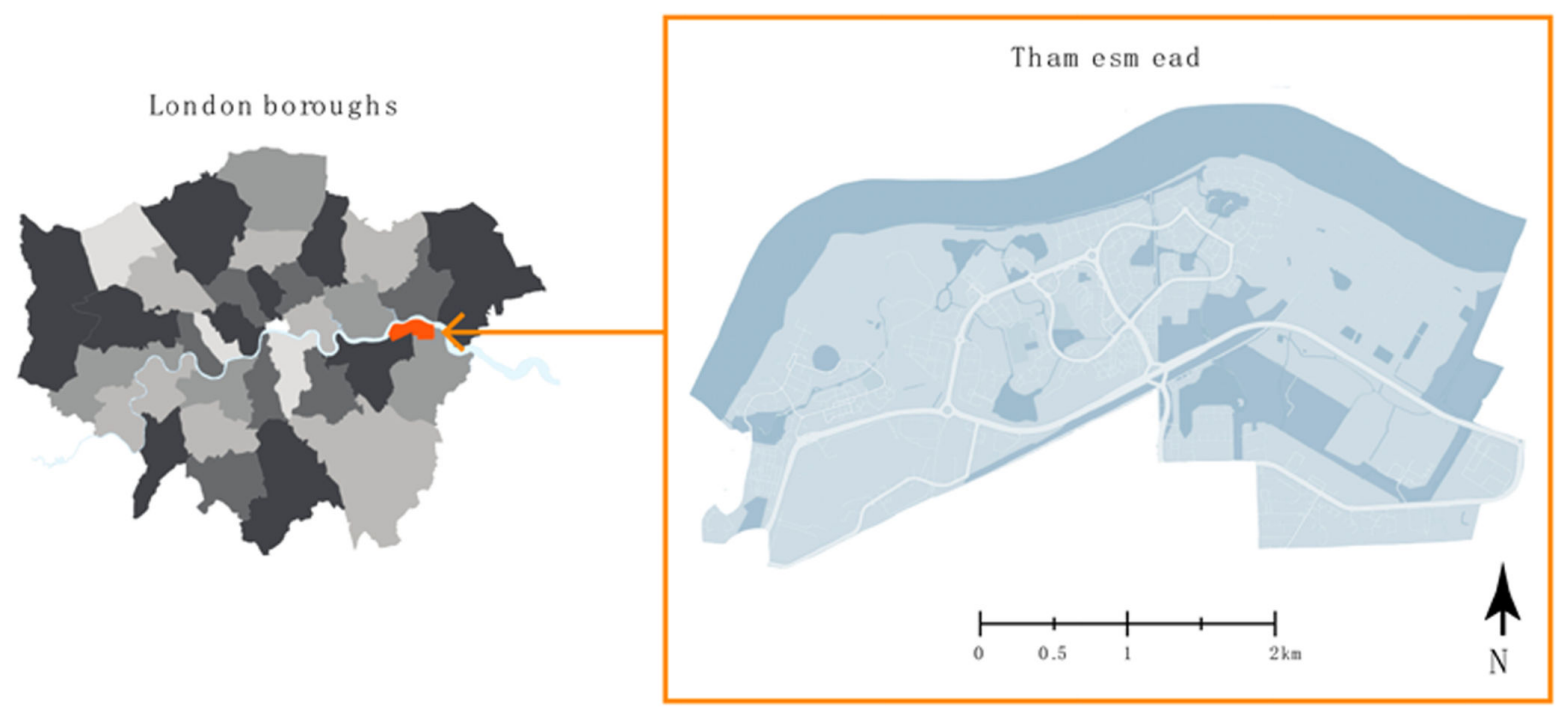
Thamesmead area location in relation to London boroughs, indicated in orange (**left**) and Thamesmead area map (**right**) (Authors).

The Thamesmead site was selected, as this paper forms part of a larger research endeavour aimed at improving residents’ and visitors’ usage of natural spaces in Thamesmead, comprising part of the CUSSH (https://projectcussh.org/, accessed on 3 July 2022) and CAMELLIA (https://www.camelliawater.org/, accessed on 3 July 2022) research projects. Thamesmead is an area located in south-east London, constituted of considerable blue and green spaces including six major lakes, seven kilometres of canals, and 75 hectares of accessible green space [34] (location detailed in Figure 3). Clearly, Thamesmead has the potential to provide its residents with ample nature contact, but currently, only 20 percent of Thamesmead residents visit the natural spaces weekly or more often [33]. With the evidenced importance of nature contact for human health and well-being, improving the usage of the natural spaces in Thamesmead is a central part of Peabody’s green infrastructure plan [33].

To identify methods of improving the usage of Thamesmead’s natural spaces, Peabody collaborated with researchers at University College London (UCL) to determine the factors that affect the use of the urban natural spaces at Thamesmead and the relationships between them. In a paper published in 2022 by Salvia et al. [19], a Systems Thinking approach was applied to data collected via Thamesmead resident interviews. The paper also outlined the relationships between urban green space usage and the multitude of factors that affect this usage [19]. Moreover, Pluchinotta et al. [42,43] focused on the Systems Thinking activities carried out with institutional stakeholders involved in the Thamesmead project who have underlined the need to focus on the use of spaces. Many factors were identified, such as accessibility, the attractiveness of the space, and socioeconomic aspects such as perceived safety and income level [42]. Within the Peabody development strategy, various biodiversity-related goals have been outlined to protect and promote a range of priority species. However, the connection between maintenance strategies, design, recreational use, and biodiversity conservation is limited. With goals outlined by Peabody to improve both the biodiversity and usage of the natural spaces at Thamesmead, we thought the Thamesmead regeneration project to be an ideal case study to test the application of the results of our CLD to a real-world project. This application shows how the CLD results can be transformed into recommendations for urban natural space design and maintenance practitioners who hope to promote both use and biodiversity in their spaces.

To bring the results of the CLD analysis into a Thamesmead-specific context, we assessed two Peabody documents. The documents are the only two publicly available documents relating to Thamesmead development plans that mention their nature- and biodiversity-related plans for the area. The first is the Living in the Landscape document [33]. This is a green infrastructure strategy encompassing Peabody’s plans for providing access to biodiverse, connected green and blue spaces for all Thamesmead residents. The second is the Thamesmead Biodiversity Action Plan [34], which complements the Living in the Landscape [33] document by specifying various habitats and species that require conservation measures and outlining steps Peabody will take to protect them.

## 3 Results

In this section, we detail the results of the CLD construction and analysis, organised into the following subsections:

1. Variables and feedback loops included in the CLD and computation of the degree centrality (DC);
2. Focus and analysis of three major interrelated feedback loops with implications for urban natural space design and policy;
3. Application of the CLD results to the Thamesmead case study.

### 3.1 Variables and Feedback Loops in the CLD

**Figure 4:**
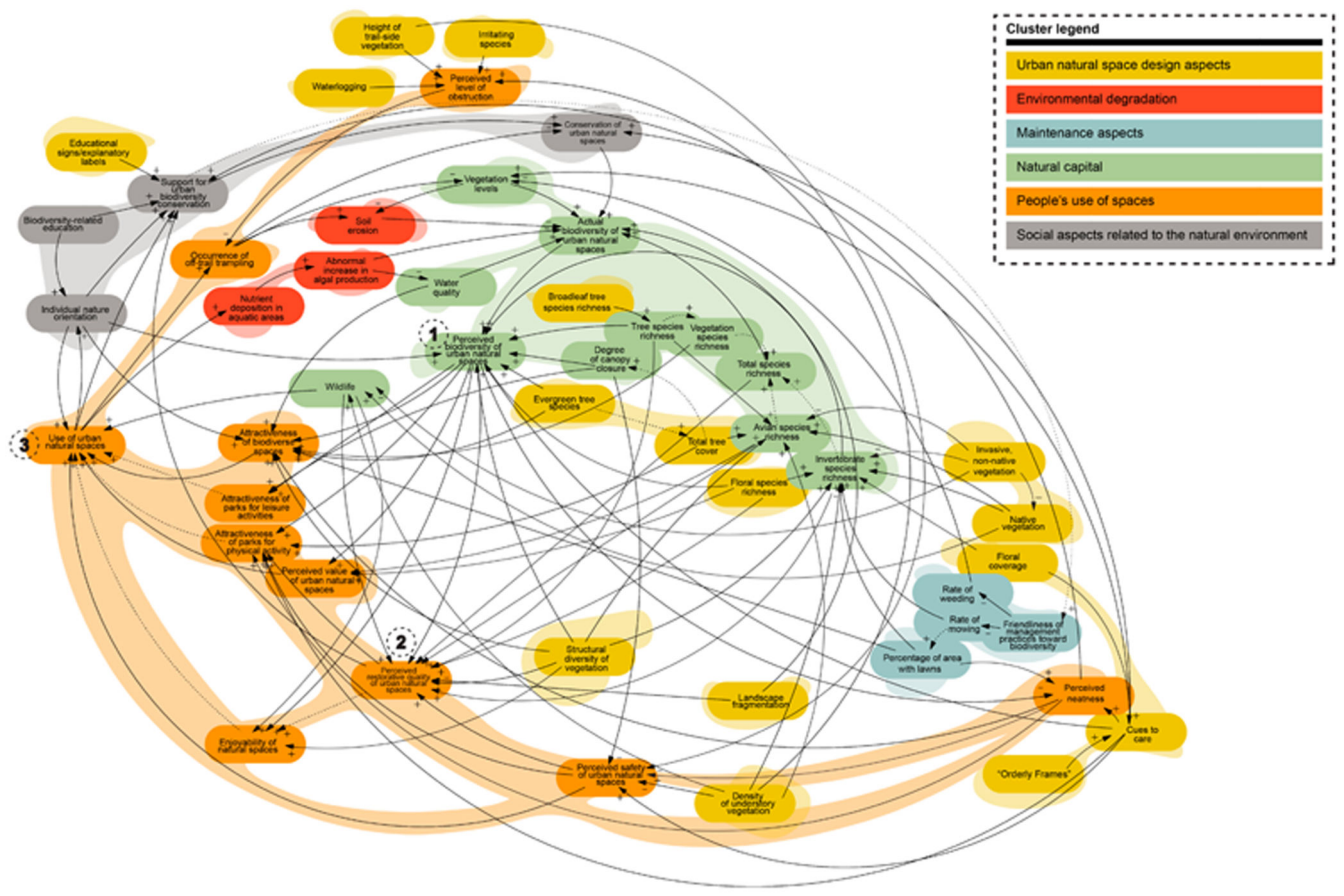
Visualisation of thematic clusters within the overall system CLD. The variables with the highest degree centrality (DC) are labelled with numbers 1–3: (1) *perceived biodiversity of urban natural spaces*; (2) *perceived restorative quality of urban natural spaces*; and (3) *use of urban natural spaces*.

The systematic literature review and open coding process produced 128 causal relationships. These relationships were implemented in the construction of a CLD, containing 49 variables and 128 links connecting them (Figure 4). A complete list of variables is provided in Appendix A. We present the variables and their connections as they were evidenced in the literature, intentionally not adding intermediary variables. The variables were categorised into thematic clusters, indicated in the legend of Figure 4. Descriptions of the thematic clusters are in Table 2. Two of the six thematic clusters encompass just over half of the variables: the design of urban natural spaces cluster (n = 13) and the natural capital cluster (n = 12). However, the natural capital cluster and the people’s use of spaces cluster are the most represented according to DC. As the focus of the analysis was on biodiversity and the use of urban natural spaces, it is logical that these are the two major clusters. Similarly, *perceived biodiversity of urban natural spaces* is the variable with the highest DC (DC = 16, Table 3). Following shortly after, at a DC value of 13, is the variable *perceived restorative quality of urban natural spaces*. Not only are these two variables the most connected, but they are also influenced by the highest number of in-arrows in the system.

There are a considerable number of loops in the CLD. For example, *use of urban natural spaces* influences *individual nature orientation*, which then influences *support for urban biodiversity conservation*, but *use of urban natural spaces* also directly influences *support for urban biodiversity conservation*. The inclusion of the intermediary variable *individual nature orientation* is important to the overall system and is directly derived from the literature.

The two variables that exist within the highest number of loops are *use of urban natural spaces* and *perceived biodiversity of urban natural spaces*, respectively. Though this finding may not be surprising, it does indicate that any change to these two variables is likely to trigger further effects throughout the system. A perhaps unexpected result was found in the variable existing within the third-highest number of loops: *support for urban biodiversity conservation*. This variable belongs to the *social aspects related to the natural environment* cluster—the only variable within this cluster to rank within the top DC variables. Existing in such a high number of loops indicates that encouraging the support of urban biodiversity conservation may have an effect on the overall system relating use and biodiversity of urban natural spaces. The interactions of this variable are assessed in Section 3.2.

### 3.2 Key Variables and Loops Influencing Use and Biodiversity

In the systematic literature review, a wide range of variables were identified that directly and indirectly affect the use and biodiversity of urban natural spaces. The literature revealed that the use of urban natural spaces has been found to increase directly with an increase in the perceived value, safety [19], and attractiveness of the space [44]. Increasing the perceived level of biodiversity directly improves the perceived value and attractiveness and thus increases the rate of usage. Unfortunately, the same increase in biodiversity indirectly reduces the perceived safety of the space through factors such as increased density of understory vegetation and the degree of canopy closure.

Sadly, with an increase in usage comes an increase in environmental degradation. According to the literature review, the major sources of environmental degradation in urban natural spaces due to recreational use are off-trail trampling and nutrient deposition in water bodies. Off-trail trampling destroys understory vegetation, which provides important habitat for some of the most essential species in an ecosystem [45]. Nutrient deposition in aquatic areas, generally due to boating or swimming, leads to abnormal levels of algae production—or eutrophication [26]. A multitude of mitigation methods have been investigated and are discussed in Sections 3.2.1–3.2.3.

This study identified three loops that play a significant role in the relationship between urban natural space use and biodiversity. The loops identified are described as follows:

1. Use, individual nature orientation, and biodiversity;
2. Perceived safety, maintenance techniques, and perceptions of biodiverse spaces;
3. Off-trail trampling, mitigation techniques, and the associated implications.
  The following three sub-sections discuss in detail these three feedback loops.

#### 3.2.1 Feedback between Use, Nature Orientation, and Biodiversity

**Figure 5:**
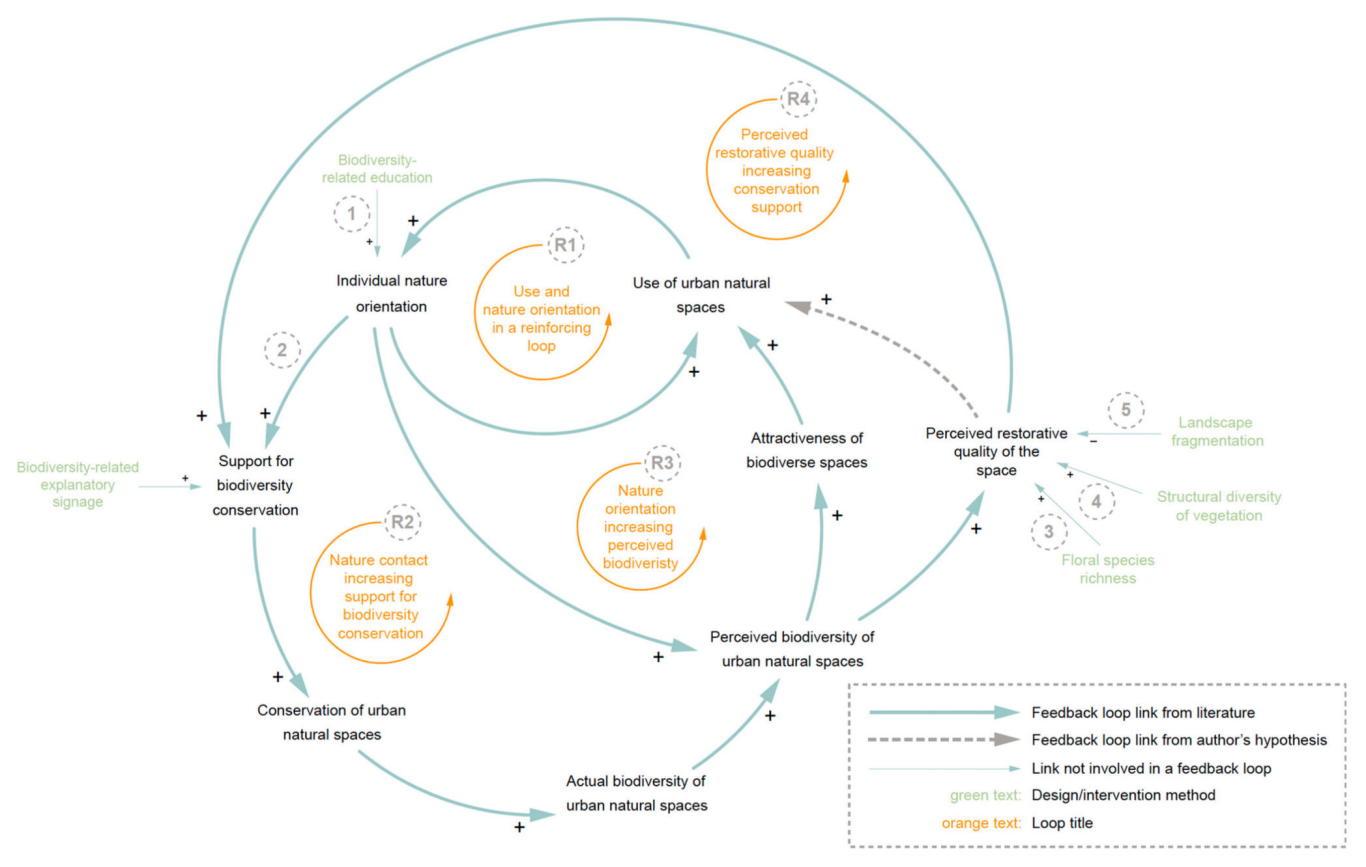
Close-up view of feedback loops surrounding use, nature orientation, and biodiversity. *Individual nature orientation* and *support for biodiversity conservation* are reinforced by use. *Perceived restorative quality of the space* further reinforces *use of urban natural spaces* and *support for biodiversity conservation*. Links referred to in the text are numbered.

Nature orientation encompasses an individual’s attitude toward the environment, with a high nature orientation indicating that a person feels a strong connection to nature and is likely to support environmental movements. Nature orientation is not a genetic factor and can be influenced by environment-related education and contact with nature [46]. An individual’s nature orientation influences many factors and is involved in various feedback loops, as detailed in Figure 5. The loops in Figure 5 are all reinforcing loops, in which the variables will continue to grow (or shrink) together until a constraining factor is introduced. Understanding these reinforcing loops provides useful insights and methods for promoting the use and biodiversity of urban natural spaces.

A simple reinforcing loop is found in loop R1 of Figure 5. The loop shows that using urban natural spaces increases an individual’s nature orientation, which in turn makes visitors more likely to visit the spaces. Not only is this loop the smallest loop in the CLD, but one of the two variables involved is a key variable in the system: *use of urban natural spaces*. Small feedback loops can indicate areas within a system where decision makers can intervene with a direct impact and minimal unintended consequences. When considering how to increase the use of urban natural spaces, this loop demonstrates that finding methods to increase people’s nature orientation will lead directly to an increase in usage. One method cited to increase an individual’s nature orientation is biodiversity-related education (Link 1) [23].

The second smallest loop in Figure 5 is loop R3, demonstrating that an individual’s nature orientation increases the level of biodiversity they perceive in a space. This, in turn, increases the attractiveness and perceived restorative quality of the space—increasing the use. Up to this point, only the perceived biodiversity of spaces has been considered, not the actual biodiversity. To bring this factor in, an important link is required: that between individual nature orientation and support for biodiversity conservation (Link 2). This relationship leads to loop R2, in which an increase in support for biodiversity conservation results in substantive conservation of urban spaces [47]. Conservation is an essential tool in allowing biodiverse spaces to thrive [48]. Loop R2 depicts how social factors like nature orientation and support for urban biodiversity can lead to tangible improvements in the measured biodiversity of urban spaces. Support for urban biodiversity can be further increased through signage in urban natural spaces, educating passers-by on nearby flora, fauna, or maintenance methods and their importance within the ecosystem.

In addition to signage, the perceived restorative quality of the space can also increase support for biodiversity conservation (loop R4) [49]. This is an important relationship to note, as the perceived restorative quality is directly affected by a few design methods and maintenance techniques. First, increasing the number of flowering species, i.e., *floral species richness*, leads to a higher perceived restorative quality (link 3) [50]. This technique engenders further beneficial factors that promote biodiversity, as depicted in Figure 5. Next, increasing the structural diversity of vegetation also evokes a higher sense of restorative quality in visitors (link 4) in addition to many other biodiversity-related benefits [51]. Structural diversity involves the complexity, arrangement, and genetic variation of vegetation structures [52]. Lastly, the level of fragmentation of a landscape has a negative impact on the perceived restorative quality of spaces. A highly fragmented space is where the natural, “wild” areas are disconnected, creating a patchy framework of habitats that hinder many species’ ability to thrive [53]. Thus, the technique that would promote the use of urban natural spaces, in this case, is connecting natural areas with nature corridors. Nature corridors are areas that provide a safe path for species to travel between natural areas.

#### 3.2.2 Feedback between Perceived Safety, Maintenance Techniques, and Biodiversity

**Figure 6:**
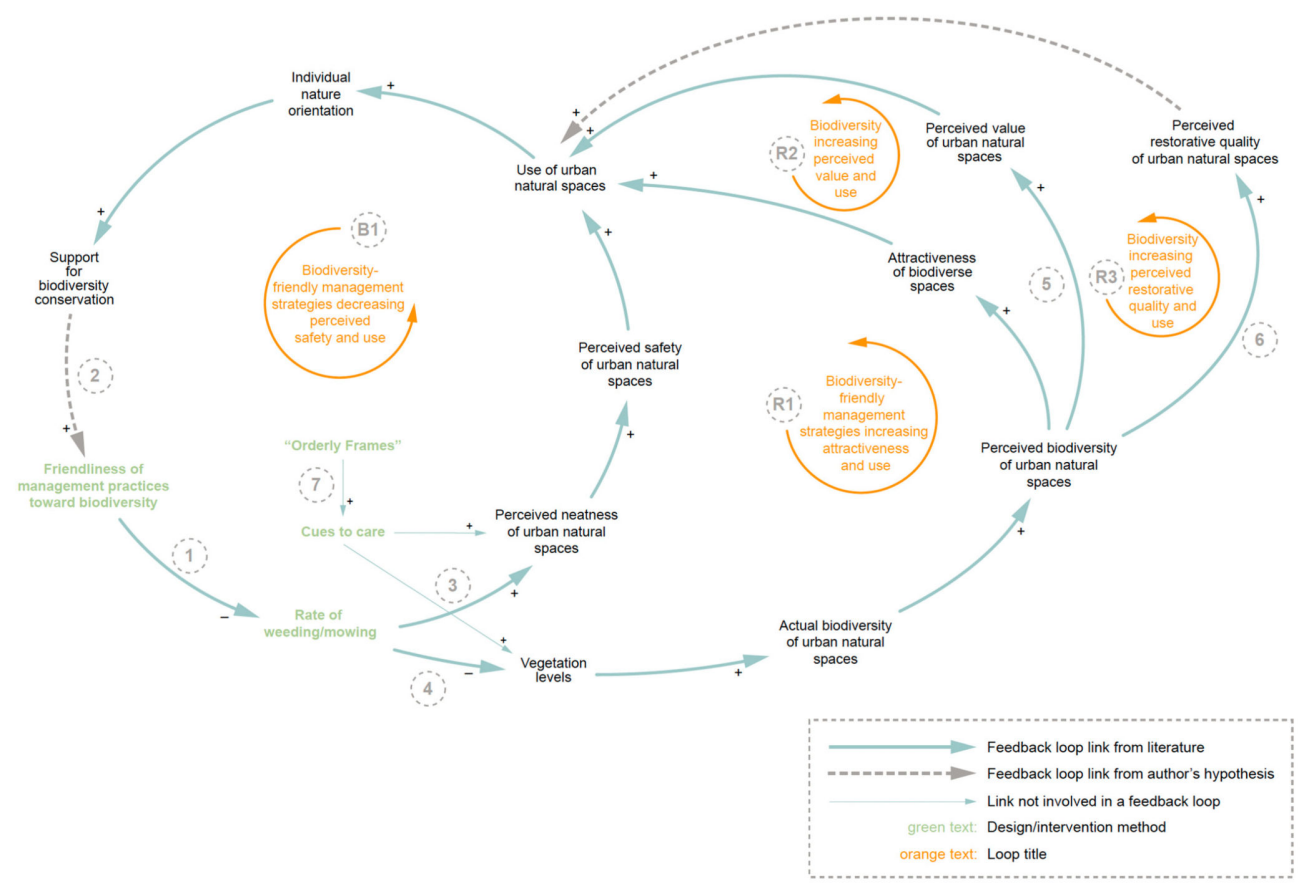
Close-up view of loops surrounding maintenance techniques and perceived safety (loop B1) and biodiversity and perceptions of spaces (loops R1, R2, and R3). Connections referred to in the text are numbered. Link 3 has been adjusted for simplification, removing the connecting variables of *percentage of area with lawns*.

Figure 6 depicts the feedback loops that result from various maintenance techniques in terms of their friendliness towards biodiversity. The friendlier a maintenance technique is towards biodiversity, the less intense it is in terms of weeding, mowing, and any other strategies that harm or reduce vegetation levels and habitat (link 1) [54]. We developed the variable *friendliness of management practices toward biodiversity*, as it provides a link to the variables involved in the *social aspects related to the natural environment* cluster. The link between *support for biodiversity conservation* and *friendliness of maintenance strategy toward biodiversity* (link 2) was hypothesised by the authors—theorising that as residents and visitors become more oriented towards nature and gather further support for biodiversity, urban natural space managers will mirror this shift. While, as expected, friendlier maintenance techniques result in higher levels of biodiversity, they also lead to the unintended consequence of a lower perceived safety of the space. This is primarily a function of the perceived neatness of the space and the density of vegetation, which is generally tied to conventional, biodiversity-harming methods of maintenance such as grass mowing, weeding, and trimming hedges (links 3 and 4) [55].

Loops R1, R2, and R3 depict the relationship between biodiverse spaces and various perceptions people hold of the spaces, resulting from the friendliness of maintenance techniques towards biodiversity. It is shown that biodiversity plays an important role in both the perceived restorative quality and the value people place on the space (Links 5 and 6). This is in addition to influencing the attractiveness of the space, as previously discussed. Hence, biodiversity levels cannot be ignored when considering factors that influence people’s use of urban natural spaces.

This result presents a conflict: One can promote biodiversity within urban natural spaces and all the associated health and environmental benefits but, in doing so, run the risk that people will feel unsafe and avoid visiting the space altogether. On the other hand, resorting to intense maintenance strategies can improve the perceived safety of a space, but these strategies greatly reduce the biodiversity of space, which would diminish the benefits associated with visiting biodiverse spaces. However, a potential mitigation solution to this conflict was cited in the literature: designing with “Orderly Frames” (link 7). Coined by Nassauer [56], “Orderly Frames” is a design principle that transforms once “messy” biodiverse spaces into purposeful areas for biodiversity promotion. This involves maintaining neat edges along trails and areas dictated for visitor use, creating a sense of intentionality in wilder areas. This is also a sign to visitors that the park is well maintained and of high quality, known as “cues to care”, also originating from Nassauer [56]. Cues to care can encompass anything that demonstrates to visitors that the park is well cared for, from signage to well-maintained trails and facilities. Incorporating cues to care can improve the perceived neatness of a space and thus improve the perceived safety. This method also promotes urban biodiversity by removing the harmful consequences of intense management strategies such as mowing and weeding. Though these concepts were conceived over twenty years ago, their effectiveness has been reiterated throughout the literature to date [57–59].

#### 3.2.3 Feedback between Off-Trail Trampling, Biodiversity, and Design and Maintenance Techniques

**Figure 7:**
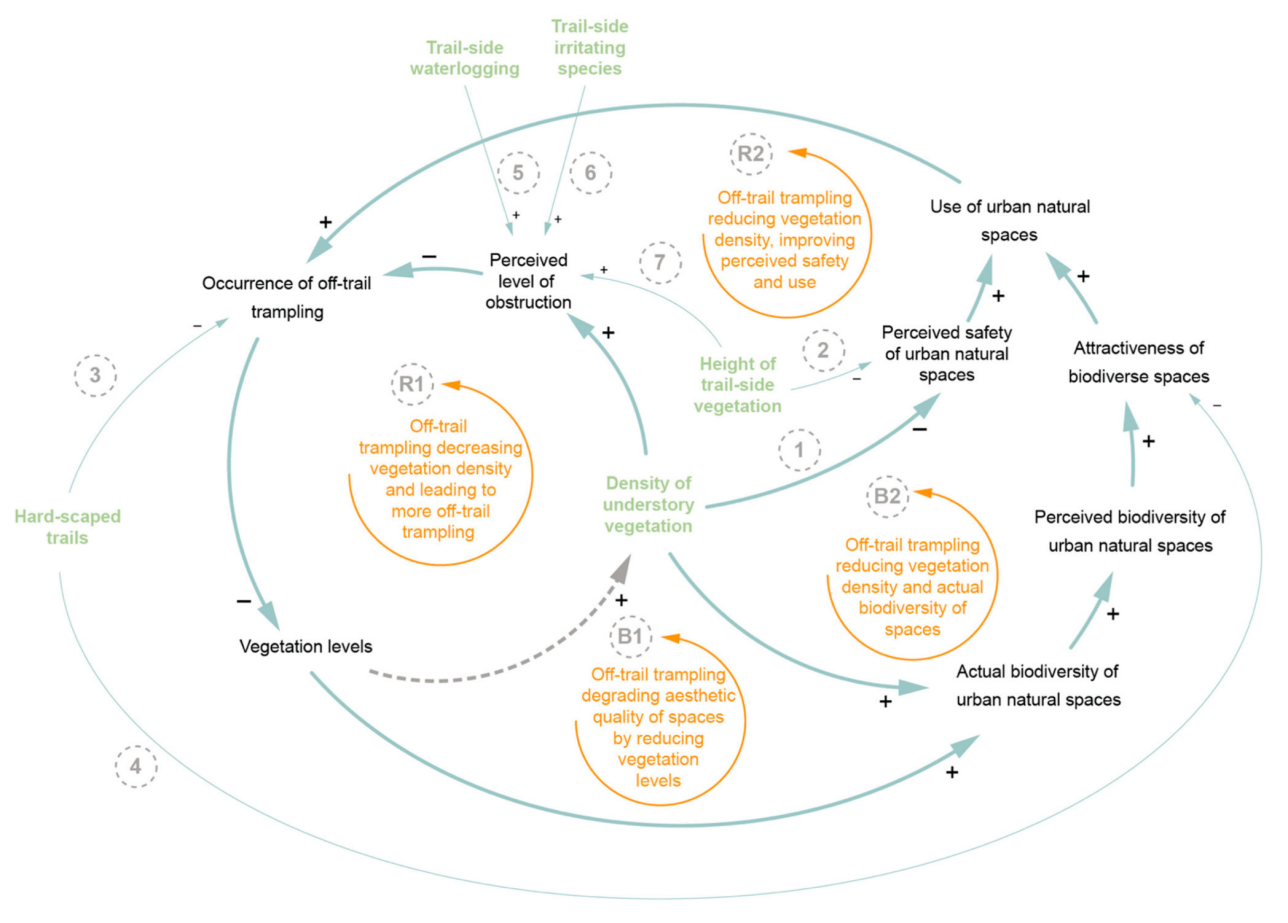
Close-up view of the effect of five design/maintenance techniques that reduce the occurrence of off-trail trampling, denoted in green. Connections referred to in the text are numbered.

Off-trail trampling has been cited as one of the most harmful recreation-related actions in terms of biodiversity conservation [45,46]. Reducing off-trail trampling is thus essential in designing biodiverse urban spaces. Figure 7 depicts the implications of various design and maintenance methods cited to reduce off-trail trampling.

The first loop that draws attention is loop R1: a reinforcing loop relating the density of understory vegetation with the occurrence of off-trail trampling. Unlike previous reinforcing loops discussed, this loop depicts a vicious cycle: As more visitors trample off-trail, vegetation levels and understory vegetation density decrease. This presents itself as a lower perceived level of obstruction, which is a major deterrent for off-trail trampling. In other words, as soon as one visitor tramples off-trail, the opportunity for trampling increases, and the likelihood that others will follow grows. Considering how harmful off-trail trampling is cited as, these results should indicate to designers and green space maintenance professionals that preventing off-trail trampling is imperative. The implications of off-trail trampling for biodiversity are depicted in loop B, showing the continual degradation of biodiversity and attractiveness of the space as off-trail trampling continues to grow.

There are many design and maintenance techniques cited to reduce the occurrence of off-trail trampling. Unfortunately, many are also associated with various negative consequences within the use-biodiversity system. For example, increasing the density of understory vegetation results in a lower perceived safety of the space (link 1) [46]. Similarly, raising the height of trail-side vegetation reduces off-trail trampling through an increased perceived level of obstruction (link 7), but it also causes the space to be perceived as less safe (link 2) [60]. Another design technique shown to prevent off-trail trampling is designing spaces with hard-scaped trails, such as wooden boardwalks or concrete paths that cannot be easily widened by trampling vegetation along the edges (Link 3) [45]. Regrettably, natural areas with trails such as these are sometimes perceived as less attractive than more natural paths such as compacted dirt (Link 4) [58].

A couple techniques have been cited to increase the perceived level of obstruction but have not yet been linked to any negative consequences for use, at least in the scope of the literature review conducted in this study. The techniques are (1) implementing marshy or waterlogged areas alongside trails (link 5) and (2) planting irritating species alongside trails, such as nettles (Link 6) [61].

#### 3.2.4 Summary of the Key Messages from the CLD Construction and Analysis

The results of the CLD analysis provide various useful and generalisable insights for understanding the relationship between urban natural space use and biodiversity. This section summarises the main messages described in the CLD analysis.

First, the importance of individual and social perceptions in relating biodiversity and use in urban natural spaces has become apparent. Encouraging the growth of individuals’ nature orientation and support for biodiversity conservation will likely lead to a greater acceptance of historically “messy” biodiverse spaces.

Next, like in any system, various trade-offs exist. The main trade-off occurs within the relationship between actual biodiversity levels and perceived safety. Various factors that promote biodiversity, such as dense vegetation, lead to a lower perceived safety of the space.

Additionally, perceived biodiversity levels lead to a lower perceived neatness and thus perceived safety in a space. While there are no solutions that can entirely mitigate this conflict, there are various design and management techniques that can reduce the negative perceptions associated with biodiverse spaces. Mainly, designing with cues to care and Orderly Frames can greatly improve the perceived safety and neatness of spaces.

Finally, the CLD highlighted the importance of preventing off-trail trampling. Without intervention, off-trail trampling enters a vicious cycle with vegetation degradation, resulting in a lower level of perceived obstruction and encouraging more off-trail trampling to occur. Urban natural spaces designers must take action to prevent off-trail trampling in natural spaces as much as possible. Various techniques can be implemented, such as designing spaces with hard-scaped trails, planting irritating species alongside trails, and encouraging denser and higher vegetation growth alongside trails to increase the perceived level of obstruction.

### 3.3 Design and Maintenance Recommendations: Application to Thamesmead, London

The literature review, alongside the construction and analysis of the CLD, allowed the study to develop insights that can be applied to the design and maintenance of urban natural spaces, specifically to the Thamesmead case study. The following section outlines recommendations that aim to mitigate various conflicts identified between use and biodiversity in urban natural spaces.

The Peabody documents focus on green space use and recreation but not on blue space recreation [33,34]. Thus, this section assesses intervention techniques aimed at improving conflicts between urban green space use and biodiversity, not blue spaces. Table 4 provides a summary of the techniques cited in the literature that have been found to mitigate issues between UGS biodiversity and use. Also noted in Table 4 is whether the technique is included in the Peabody documents as well as the technique’s location with the CLD analysis.

Four out of the twelve intervention techniques resulting from this study are included in the Peabody documents analysed (rows 1–3, 8, Table 4). These include reducing the rate of mowing and weeding, providing biodiversity-related education, and connecting natural sites with vegetated corridors. While the latter two were not found to have any negative consequences in the CLD analysis, reducing the rate of mowing and weeding was shown to reduce the perceived neatness and thus perceived safety and attractiveness of the space. To mitigate these effects, it is recommended that Peabody design the Thamesmead natural spaces with Orderly Frames, providing cues to care in the space (rows 9–10, Table 4). In a systematic literature review conducted by [62], it was found that for areas with wildly grown vegetation to be accepted by urban residents, a noticeable and beneficial human influence is essential. Not only will this improve the perceived safety of the spaces, but providing cues to care can also increase visitors’ wider support for biodiversity conservation [56]. Further guidelines for designing with cues to care can be found in Nassauer’s “Messy Ecosystems, Orderly Frames” [56] and in Hoyle et al.’s “What determines how we see nature? Perceptions of naturalness in designed urban green spaces” [58].

Biodiversity-related educational signage can be seen as “cues to care” by informing residents that a “messy” natural space is purposefully messy to promote biodiversity [56]. Biodiversity-related education also plays a large role in promoting the usage of urban natural spaces by increasing individuals’ nature orientation [23]. Educational programs that provide residents with information surrounding the importance of biodiversity conservation and what that may look like in their neighbouring natural space will likely create a more positive and accepting attitude toward “messier” nature [46].

Though cited as a major source of biodiversity degradation in natural spaces in the literature, off-trail trampling is not mentioned in either of the Peabody documents. When introducing trails into natural habitats—as is involved in Peabody’s plans for Thamesmead—it is imperative for biodiversity conservation that off-trail trampling be prevented as much as possible, especially around sensitive habitats. In the literature review, five main techniques were found as proven methods for effectively reducing off-trail trampling (rows 2, 4–7, Table 4). One out of the five was shown to directly reduce off-trail trampling: establishing hard-scaped trails [63]. The other four reduce off-trail trampling by way of increasing the perceived level of obstruction alongside the trail, such as tall trail-side vegetation (at least 54 cm tall), dense understory vegetation, marshy or waterlogged trailside areas, and irritating trail-side vegetation [46,61]. However, for the most ecologically sensitive areas, it has been recommended to either reduce the number of paths leading to that area or prohibit access entirely [46]. For the former option, recreational paths and areas with high levels of use are recommended to be located in less ecologically sensitive areas, known as a “confinement’” strategy [25]. Again, it is important to keep in mind that most factors are associated with various trade-offs. For example, when implementing hard-scaped trails, this may be associated with a lower perceived attractiveness that is linked to the presence of unnatural or human-made elements in natural areas [58].

Lastly, one of the most influential factors identified in the CLD construction and analysis was the perceived restorative quality of urban natural spaces. This influences factors such as the attractiveness of parks for physical activity, the enjoyability of spaces, and even the support of biodiversity conservation. It is also influenced by many factors that are directly controlled by designers and maintenance professionals, with the added benefit of also promoting biodiversity. Three factors are primarily cited in the literature: increasing floral species richness and coverage [50], increasing the structural diversity of vegetation [51], and reducing landscape fragmentation [64].

## 4 Discussion

This study has provided the first application of a Systems-Thinking modelling approach directed at understanding the specific relationship between urban natural space use and biodiversity. Many themes established in the study mirror those of a System-Dynamics case study conducted by Salvia et al. [19] surrounding the use of natural spaces, with application in Thamesmead. Nevertheless, the results fill a knowledge gap identified by the current authors, among other studies [19,20], to further investigate the interconnections between factors surrounding urban natural space use and biodiversity.

This study, by developing a CLD from causal relationships derived from a systematic literature review, uncovered interrelationships that are under-explored and underemphasised in the urban natural space design and maintenance sphere. The study further demonstrated the benefits associated with applying a Systems-Thinking modelling approach to understand various factors related to urban natural space use, complementing the findings of Salvia et al. [19]. This section covers the findings of the research in relation to current studies and urban natural space design and maintenance practices. The results are compared with previously cited relationships and factors associated with the biodiversity and use of urban natural spaces, highlighting the importance of individual and social values in the system. The practical implications deriving from the study are highlighted throughout.

The findings from the CLD constructed during this study indicate that the factors relating urban natural space use and biodiversity are widespread, diverse, and heavily influenced by individual and social perceptions. Some findings align with and reinforce current ideologies; others highlight a recent contradiction of various long-held beliefs. The major variables involved in this phenomenon are discussed in this section, drawing insights from the past literature on trends in various perceptions.

Firstly, perceived restorative quality of the space both influences use and is greatly influenced by various biodiversity-related factors. This is demonstrated by the variable’s high DC value (DC = 13) and level of connectivity (no. loops = 221) in the CLD. It also is consistently cited across the decades through several studies such as those conducted by Jorgen and Gobster [57], Houlden, Jani and Hong [50], Costigan et al. [65], and Fisher et al. [49]. Thus, urban natural space designers and management practitioners who are interested in increasing use and biodiversity may increase the perceived restorative quality of their spaces. There are many methods to do this; however, the two main methods deriving from this study that also directly increase the biodiversity of the space are (1) increasing the coverage and diversity of native flowering species and (2) increasing the structural diversity of plantings (tall, medium, and low vegetation and tree heights).

Another group of perceptions outlined in the literature over two decades before this study still appear to hold true and play an important role in the relationship between urban natural space use and biodiversity. This group includes perceived safety [66] and perceived neatness [56]. The relationship between “fear” or perceived safety and various biodiversity-related factors still appears to ring true, with a higher level of biodiversity resulting in a lower perceived safety of the space (see Section 3.2.2). However, this study identified the specific factors that define the relationship between biodiverse spaces and perceived safety, allowing these factors to be addressed with design and maintenance measures rather than simply used in an argument against bringing biodiversity into urban natural spaces. Relationships between both the density of understory vegetation [46,60] and the perceived neatness [67] of a space have been repeatedly cited to negatively affect perceived safety. These two factors are closely related to biodiversity levels, with a high density of understory vegetation required to promote biodiversity [54] and a lower perceived neatness resulting from highly biodiverse spaces [58]. Designing with Orderly Frames and cues to care, which is detailed in Section 3.2.2, can greatly improve the perceived neatness of a space. The density of understory vegetation is a complex variable in the system: While it does decrease perceived safety, it is also a major variable in discouraging off-trail trampling—the biggest identified use-related factor that directly reduces biodiversity. These relationships represent trade-offs that are common to every system [27]. The current literature around these relationships does not provide sufficient information for practitioners on how to best balance these variables. Future research could investigate these relationships, and further guidance is provided in Section 5.

Various ideologies do, however, appear to be losing their footing, with positive implications for acceptance of biodiverse spaces. Up until the 1990s, it was cited that “wild”, unmanicured spaces not only result in a lower perceived attractiveness but that they might lead to visitors experiencing fear and even disgust [68]. However, through the systematic literature review and CLD construction, it has become clear that this ideology is, for the most part, outdated. The perceived level of biodiversity has been concretely linked to a higher level of attractiveness [58] and an increased perceived restorative quality of the space [24]. These results are far from “disgust”. Additionally, visitors are more likely to engage in both physical and leisure activities in spaces with a higher perceived level of biodiversity [22,23]. All these factors positively influence the use of urban natural spaces, with the perceived level of biodiversity found to have the highest DC (DC = 16) within the CLD. These results provide further incentive for urban natural space designers and management practitioners to promote biodiversity in their spaces.

Another ideology that appears to have lost some footing is the relationship between excessive and unknown wildlife and perceived safety [66]. Not only has the presence of wildlife been linked to a high perceived restorative quality of the space [7] and enjoyability of the space [69], but it has been directly linked to an increased likelihood that people will visit the space [70]. The cumulative effect of these results indicates that public attitudes may indeed be shifting towards a more biodiversity-accepting and conservation-supporting atmosphere, as Southon et al. [71] hypothesised. This shift is mainly driven by individuals’ orientation towards nature [72].

While several studies have cited the importance of contact with nature in increasing individuals’ nature orientation [17,73,74], there has been little research conducted surrounding active methods for encouraging the growth of people’s nature orientation. Fischer et al. [23] and Kowarik [72] noted that providing biodiversity-related education may increase one’s nature orientation. Unfortunately, another study found that providing education only increased visitors’ support for biodiversity conservation if they had a high existing nature orientation [75]. In the end, with the information at the time of this study, designing and maintaining urban natural spaces to encourage the highest rate of usage and thus contact with nature is the most concrete action known to increase people’s nature orientation.

## 5 Conclusions

This study has uncovered important interrelationships between urban natural space biodiversity and use that are applicable to the design and management of these spaces on both a global and local scale. This was achieved by constructing a CLD with data derived from a systematic literature review, allowing knowledge gathered on the topic across the globe to be captured and visually mapped. In this case, the application of Systems Thinking generated many valuable insights. First, the causal map highlighted the significant role that individual and social values and perceptions play in relating urban natural spaces use and biodiversity. People’s perceptions of biodiverse spaces are highly dependent on their individual nature orientation, i.e., how much they care for the environment. This also influences their likelihood of supporting and promoting biodiversity conservation projects. Researching and developing methods that encourage the growth of residents’ nature orientation, such as biodiversity-related education, will ultimately lead to a higher acceptance of and demand for biodiverse spaces. Second, the CLD revealed some insights into the relationship between perceived safety and biodiversity. While some long-held beliefs were shown to be outdated—such as universal aesthetic preference for highly manicured and maintained natural spaces, which, in places, has shifted to a preference for wilder natural spaces—other concepts still appear to ring true. These include the association of a lower perceived safety with both dense vegetation and a lower perceived neatness. It is recommended that designers implement Orderly Frames and cues to care in their design methodology to improve the perceived safety and neatness of spaces while still allowing biodiversity to thrive. Finally, the importance of preventing off-trail trampling was demonstrated in the CLD analysis. Without measures in place to discourage it, its occurrence will continue to grow alongside vegetation trampling, as vegetation acts as a barrier for off-trail trampling. Recommended measures to prevent off-trail trampling include planting trail-side irritating species, increasing the height of trailside vegetation, implementing marshy areas alongside trails, and establishing “hardened” trails with materials such as wood or concrete. However, these measures are associated with various trade-offs, such as reducing perceived safety and perceived restorative quality, and further research could investigate how to balance these. For example, further research could investigate the relationship between understory, off-trail trampling, and perceived safety. The research might attempt to identify a level of understory vegetation that sufficiently discourages off-trail trampling while not having a negative influence on perceived safety, one of the main trade-offs identified in the system. Another interesting direction of future research could be investigating the relationship between urban natural space use and presence of irritating species, as this would provide valuable information on this method’s success in both protecting biodiversity and promoting use.

On a local scale, the CLD analysis was able to assess Peabody’s Thamesmead development plans and provide various recommendations, providing a useful example of the application of the CLD results in a localised context. However, there exists a limitation in this application in that the literature analysed for the systematic literature review included papers and experiments conducted across the globe. Considering that the topic of many studies involved the individual perceptions of people—which vary greatly between countries and cultures—this posed a large limitation in the application of the results to a specific site such as Thamesmead. The individual preferences of the Thamesmead residents likely differ to some extent from the findings of this analysis. Incorporating a participatory process wherein relevant stakeholders aide in the construction or validation of the CLD would have further strengthened the results, indicating an area of further research in this area. Future research could take the generalisable CLD and apply it to a case study, involving relevant stakeholders in the CLD validation process. However, as a contribution to the global research space of natural space use and biodiversity, the results provide legitimate insights into the interrelationships and key components to be considered by designers and maintenance professionals, which should always be placed in a local, site-specific context through participatory engagement processes.

In addition to the many areas already highlighted by this study for potential future research, there are a few others to which we would like to call attention. Firstly, given the importance and interconnectedness of individual nature orientation in this area, future research could focus on methods of encouraging the growth of individuals’ nature orientation and the result this growth would have on the wider system. Next, while not directly relevant to the Thamesmead case study, investigating the differences in use of these spaces by different types of users based on diversity metrics or whether they are long- vs. short-term residents or residents vs. tourists would be an important development direction in the general research sphere. Finally, while this study has provided further theoretical understanding and guidance for design and maintenance professionals of the trade-offs between biodiversity and urban natural space use, further work is needed in developing actionable and practical frameworks and guidance for urban natural space design, ideally on an urban landscape archetype scale (e.g., greenways, neighbourhood parks, waterfronts, etc.).

## Supplementary Material

Supplementary Materials

## Figures and Tables

**Table 1:** Key words applied in literature search. Asterisk (*) refers to words where the search includes any extension of the word, e.g.,: Urban green * returns urban green, urban greenspace, urban greening, etc.

	Urban Natural Space	Biodiversity	Use
Search terms	Urban green * Park * Urban blue *	Biodivers *	Use
Location	Title, Abstract, Keywords	Title, Abstract, Keywords	Title, Abstract, Keywords

**Table 2:** Descriptions of thematic clusters implemented in the CLD.

Cluster	Description
Urban natural space design aspects	This cluster includes any aspect of urban natural spaces that can be directly altered or influenced by designers. This included the design of trails and other manmade aspects to natural elements such as the species richness of planted trees and flowering species.
Environmental degradation	Variables that involve any direct environmental degradation, in either direction of growth or shrinkage, are included in this cluster. An example is soil erosion, which degrades the environment regardless of the rate of erosion.
Maintenance aspects	Any variables that involve decisions made by urban natural space maintenance professionals, including management aspects such as the rate of mowing and weeding, are included in this cluster.
Natural capital	This cluster includes any variables related to natural resources that cannot be directly influenced by either maintenance or design measures. This includes factors such as animal and invertebrate species richness and biodiversity.
Social aspects related to the natural environment	This thematic cluster includes any variable that relates to social issues and preferences toward the natural environment. This included biodiversity-related education and support for biodiversity conservation.

**Table 3:** Variables with the highest degree centrality (DC) and their associated number of in- and out-arrows.

Variable	Out-Arrows	In-Arrows	Total No. of Connections	No. of Loops	Cluster
*Perceived biodiversity of urban natural spaces*	7	9	16	399	Natural capital
*Perceived restorative quality of urban natural spaces*	3	10	13	221	People’s use of spaces
*Use of urban natural space*	4	8	12	411	People’s use of spaces
*Invertebrate species richness*	3	8	11	192	Natural capital
*Attractiveness of biodiverse spaces*	1	8	9	76	People’s use of spaces
*Actual biodiversity of urban natural spaces*	1	7	8	313	Natural capital
*Avian species richness*	2	6	8	0	Natural capital
*Support for urban biodiversity conservation*	2	6	8	344	Social aspects related to the natural environment
*Cues to care*	5	3	8	0	Urban natural space design aspects
*Perceived safety of urban natural spaces*	3	5	8	121	People’s use of spaces

**Table 4:** Techniques that address a conflict/relationship between biodiversity and UGS use, specific to the Peabody Thamesmead documents. Location of the technique in the CLD analysis is noted.

No.	Biodiversity/Use Relationship	Biodiversity-Promoting Design/Maintenance Technique	Included in Peabody Documents?	Location in CLD Analysis
1	High rate of mowing leads to lower vegetation levels and invertebrate species richness.	Reduce frequency and extent of mowing	Yes	Figure 6, loops R1, R2, and R3
2	Off-trail trampling degrades vegetation density, and high rate of weeding reduces important habitat and overall biodiversity.	Reduce weeding/encourage dense understory vegetation growth	Yes	Figure 5, loops R1 and B2
3	Low nature orientation reduces support for and implementation of biodiversity conservation projects.	Provide biodiversity-related education	Yes	Figure 5, link 1
4	Off-trail trampling degrades vegetation levels and overall biodiversity.	Increase height of trail-side vegetation to increase perceived obstruction	No	Figure 7, link 7
5	Off-trail trampling degrades vegetation levels and overall biodiversity.	Plant trail-side irritating species to increase perceived obstruction	No	Figure 7, link 6
6	Off-trail trampling degrades vegetation levels and overall biodiversity.	Implement waterlogged/marshy areas alongside trails to increase perceived obstruction	No	Figure 7, links 3 and 4
7	Off-trail trampling degrades vegetation levels and overall biodiversity.	Establish hard-scaped trails	No	Figure 7, links 3 and 4
8	Landscape fragmentation reduces connectivity and reduces perceived restorative quality of the space.	Connect natural sites with vegetated corridors	Yes	Figure 5, link 5
9	Biodiverse spaces are often perceived as messy, reducing the perceived safety and attractiveness of the space.	Cues to care provide explanations for biodiverse spaces and increase the acceptance of “messy” biodiverse spaces	No	Figure 6, link 6
10	Biodiverse spaces are often perceived as messy, reducing the perceived safety and attractiveness of the space.	Orderly Frames outline biodiverse spaces, allowing habitats to thrive while also providing access to people	No	Figure 6, link 6
11	Perceived restorative quality of spaces greatly influences use of urban natural spaces.	Increasing the coverage and diversity of native flowering species increases the perceived restorative quality	No	Figure 5, link 3
12	Perceived restorative quality of spaces greatly influences use of urban natural spaces.	Increasing the structural diversity of plantings (tall, medium, and low height vegetation and trees) increases the perceived restorative quality	No	Figure 5, link 4

## Data Availability

Data is contained within the article.
