# Supplementary material for: Interrelationships and Trade-Offs between Urban Natural Space Use and Biodiversity
Source: Sustainability. Author manuscript; Available in PMC 2024 Oct 16. (PMC7616704; doi:10.3390/su16104051)
Supplement: Supplementary Materials [file EMS199302-supplement-Supplementary_Materials.pdf]

Appendix A

This appendix provides a list of the variables implemented in CLD, their definitions, and their associated references.

Table A1. Variables included in the CLD, their definitions, and references.

Variable	Definition	Reference
Abnormal increase in algal production	An increase in production of algae that is above normal level for the specified ecosystem	[26]
Actual biodiversity of urban natural spaces	The objective level of biodiversity in an urban natural space, measured via an accepted metric such as species richness, species dominance, or species presence	[76]
Attractiveness of biodiverse spaces	An individual's personal preference toward the visual and aesthetic quality of spaces with high biodiversity	[77]
Attractiveness of parks for leisure activities	The attractiveness of an urban green space to engage in leisure activities, such as reading, sitting, and other low-intensity activities	[22]
Attractiveness of parks for physical activities	The attractiveness of an urban green space to engage in physical activity, linked to the likelihood a person would engage in physical activity in that space	[65]
Avian species richness	The number of bird species within a defined region, as defined by the observer	[78]
Biodiversity-related education	The occurrence of any type of activity that raises awareness or increases understanding of biodiversity-related topics	[23]
Butterfly species richness	The number of butterfly species within a defined region, as defined by the observer	[78]

Table A1. *Cont.*

Variable	Definition	Reference
Conservation of urban natural spaces	The conservation of natural spaces from urban development, preservation of their natural elements from forms of destruction	[48]
Cues to care	Cues within urban natural spaces that show some type of human intervention with highly biodiverse spaces; some examples include pathways, neat edges, and signage	[56]
Degree of canopy closure	A measure of how much light is allowed to enter through a set of trees' canopies; a high degree of canopy closure is associated with a low amount of light	[46]
Density of understory vegetation	An indication of the density of vegetation, i.e., how tightly knit plants are to each other, that lies on the understory, i.e., ground level, of a green space.	[54]
Educational signs/explanatory labels	Presence of signs that describe or provide an explanation of the method of maintenance or area and how it promotes biodiversity	[23]
Enjoyability of natural spaces	The perceived enjoyability an individual associates with the specified natural space; enjoyability is a subjective feeling reported by visitors via survey or similar method	[79]
Evergreen tree species	This variable describes the presence of tree species that are classified as evergreens; as the variable grows, so does the number of evergreen tree species in the specified area; evergreens do not shed their leaves in any season but remain green and functional throughout the year	[80]
Floral coverage	The percentage of the specified area that is covered by flowering plants	[71]
Floral species richness	The number of flowering species within a defined region, as defined by the observer	[78]
Friendliness of management practices toward biodiversity	The level that management practices promote biodiversity and ecosystem health	Defined by authors
Height of trail-side vegetation	The height of vegetation immediately bordering a trail	[61]
Individual nature orientation	An individual's attitude toward the environment: A high nature orientation would indicate that an individual feels a strong connection to nature and would care heavily about protecting the environment; a person with a low nature orientation would not feel strong emotions toward nature, e.g., a feeling of indifference	[81]
Invertebrate species richness	The number of invertebrate species within a defined region, as defined by the observer	[78]
Trail-side irritating species	The planting of irritating species along the sides of trails; irritating species are any type of vegetation that cause irritation among humans, such as stinging nettle or poison ivy	[61]
Landscape fragmentation	Landscape fragmentation is measured by the number and size of patches of natural vegetation; for example, a large number of small patches is associated with a high level of landscape fragmentation	[64]
Native vegetation	Percentage share of total vegetation that is native to the region, as detailed by local environmental authorities	[82]
Nutrient deposition in aquatic areas	The level of nutrients deposited into a specified aquatic area over a specified temporal scale; sources of nutrient deposition include sediment re-suspension and direct nutrient additions from soaps, detergents, sunscreen, and biological wastes	[26]
Occurrence of off-trail trampling	The occurrence of any travelling by visitors that is not on an official trail as specified by the space management team or designers	[25]
Orderly Frames	A design methodology developed by Nassaeur that involves creating neat edges around biodiverse spaces to improve the perceived neatness and accessibility of the space; further guidelines for implementation can be found in Nassaeur's paper [56]; an increase in this variable is associated with a design closer to that outlined by Nassaeur	[56]

Table A1. *Cont.*

Variable	Definition	Reference
Perceived biodiversity of urban natural spaces	A measure of an individual's perceived level of overall biodiversity, measured via qualitative measures such as questionnaires	[64]
Perceived level of obstruction	A measure of an individual's perceived level of obstruction in a specified area, measured via qualitative measures such as questionnaires	[61]
Perceived neatness of urban natural spaces	A measure of an individual's perceived level of neatness in a specified area, measured via qualitative measures such as questionnaires	[58]
Perceived restorative quality of urban natural spaces	A measure of an individual's perceived level of the restorative effect received by visiting a specified area, measured via qualitative measures such as questionnaires	[65]
Perceived safety of urban natural spaces	A measure of an individual's perceived level of personal safety experienced when visiting a specified area, measured via qualitative measures such as questionnaires	[60]
Perceived value of urban natural spaces	A measure of an individual's perceived level of value placed on a specified area, measured via qualitative measures such as questionnaires; value is defined as the worth and usefulness of the space	[83]
Percentage area with lawns	The percentage of a specified area that is covered by lawns, which are defined as any area covered with grass and maintained by mowing	[55]
Rate of mowing	The rate of mowing activities in terms of occurrences over a specified time period	Defined by authors
Rate of weeding	The level of weeding activities in terms of vegetation removed	Defined by authors
Soil erosion	The loss of soil from an area due to various ecological processes	[84]
Species diversity of broadleaf tree species	The diversity of leaf forms between various broadleaf tree species	[64]
Structural diversity of vegetation	The variation within the structure of vegetation, which includes the complexity, arrangement, and genetic variation within the vegetation	[52]
Support for urban biodiversity conservation	This encompasses any positive attitude toward biodiversity conservation projects or movements, as defined by individuals via surveys or through action	Defined by authors
Total species richness	The total number of species within a defined region, as defined by the observer	[78]
Total tree cover	The percentage of a specified area that is covered by trees; this is generally measured via satellite, including the tree canopy as coverage	[85]
Tree species richness	The number of unique tree species within a defined region, as defined by the observer	[78]
Use of urban natural spaces	Any type of visit to an urban natural spaces, regardless of the purpose or length of visit	[18]
Vegetation levels	The amount of vegetation in a specified space, as measured by the observer	[25]
Vegetation species richness	The number of vegetative species within a defined region, as defined by the observer	[78]
Water quality	This variable refers to the biological, physical and chemical characteristics of water, depending on the standards of its usage or purpose; for natural waters, this standard can be defined as its natural quality without degradation or intervention from humans, and a decrease in this variable is associated with a poorer quality of water	[86]
Trail-side waterlogging	The implementation of waterlogged or marshy areas along trail sides	[61]
Wildlife	The presence of any species within the animal kingdom in an area	[87]

Appendix B

This appendix presents the interrelationships between variables used to construct the CLD as well as their associated references.

Table A2. Causal relationships and their associated references.

Cause	Effect	+/−	Reference
Abnormal increase in algal production	Water quality	−	[86]
Abnormal increase in algal production	Actual biodiversity of urban natural spaces	−	[26,88]
Actual biodiversity of urban natural spaces	Perceived urban natural space biodiversity	+	[51]
Attractiveness of biodiverse spaces	Use of urban natural spaces	+	[42]
Attractiveness of parks for leisure activities	Use of urban natural spaces	+	Hypothesised by authors
Attractiveness of parks for physical activities	Use of urban natural spaces	+	Hypothesised by authors
Avian species richness	Perceived restorative quality of the space	+	[49,64]
Avian species richness	Perceived value of urban natural spaces	+	[83]
Avian species richness	Total species richness	+	Hypothesised by authors
Biodiversity-related education	Nature orientation	+	[23]
Biodiversity-related education	Support of urban biodiversity conservation	+	[46,59,71]
Conservation of urban natural spaces	Actual biodiversity of urban natural spaces	+	[48]
Cues to care	Perceived safety	+	[66]
Cues to care	Perceived neatness of urban natural spaces	+	[58]
Cues to care	Perceived urban natural space biodiversity	−	[58]
Cues to care	Attractiveness of biodiverse spaces	+	[57]
Cues to care	Conservation of urban natural spaces	+	[56]
Cues to care	Vegetation levels	+	[56]
Degree of canopy closure	Perceived urban natural space biodiversity	+	[62]
Degree of canopy closure	Perceived safety	−	[46]
Degree of canopy closure	Wildlife	+	[89]
Degree of canopy closure	Attractiveness of parks for leisure actives	+	[90]
Density of understory vegetation	Perceived level of obstruction	+	[46]
Density of understory vegetation	Perceived safety	−	[46]
Density of understory vegetation	Avian species richness	+	[54]
Density of understory vegetation	Invertebrate species richness	+	[54]
Density of understory vegetation	Wildlife	+	[54]
Density of understory vegetation	Perceived safety	−	[60]
Diversity of broadleaf tree species	Tree species richness	+	[64]
Educational signs/explanatory labels	Support of urban biodiversity conservation	+	[74]
Enjoyability of natural spaces	Use of urban natural spaces	+	Hypothesised by authors
Evergreen tree species	Attractiveness of biodiverse spaces	+	[91]
Evergreen tree species	Perceived restorative quality of the space	+	[64]
Floral coverage	Cues to care	+	[58]
Floral coverage	Invertebrate species richness	+	[92]
Floral species richness	Perceived restorative quality of the space	+	[50]
Floral species richness	Perceived urban natural space biodiversity	+	[58]
Floral species richness	Perceived value of urban natural spaces	+	[71]
Friendliness of management practices toward biodiversity	Rate of mowing	−	[54]
Friendliness of management practices	Rate of weeding	−	[54]

Table A2. *Cont.*

Cause	Effect	+/−	Reference
Hard-scaped trails	Occurrence of off-trail trampling	−	[63]
Height of trail-side vegetation	Perceived level of obstruction	+	[61]
Height of trail-side vegetation	Perceived safety	−	[60]
Invasive, non-native vegetation	Avian species richness	−	[93]
Invasive, non-native vegetation	Invertebrate species richness	−	[93]
Invasive, non-native vegetation	Native vegetation	−	[93]
Invasive, non-native vegetation	Wildlife	−	[93]
Invertebrate species richness	Perceived restorative quality of the space	+	[64]
Invertebrate species richness	Total species richness	+	Hypothesised by authors
Invertebrate species richness	Perceived urban natural space biodiversity	+	[58]
Irritating species	Perceived level of obstruction	+	[61]
Landscape fragmentation	Perceived restorative quality of the space	−	[64]
Landscape fragmentation	Actual biodiversity of urban natural spaces	−	[53]
Native vegetation	Avian species richness	+	[54]
Native vegetation	Invertebrate species richness	+	[54]
Native vegetation	Wildlife	+	[54]
Nature orientation	Attractiveness of biodiverse spaces	+	[24]
Nature orientation	Perceived urban natural space biodiversity	+	[58]
Nature orientation	Support of urban biodiversity conservation	+	[72,94]
Nature orientation	Use of urban natural spaces	+	[44]
Nutrient deposition in aquatic areas	Abnormal increase in algal production	+	[26]
Occurrence of off-trail trampling	Soil erosion	+	[45]
Occurrence of off-trail trampling	Vegetation levels	−	[45]
Occurrence of off-trail trampling	Conservation of urban natural spaces	+	[46]
Orderly Frames	Cues to care	+	[56]
Perceived biodiversity of urban natural spaces	Attractiveness of biodiverse spaces	+	[21]
Perceived biodiversity of urban natural spaces	Attractiveness of parks for physical activities	+	[23]
Perceived biodiversity of urban natural spaces	Perceived restorative quality of the space	+	[49,58,64]
Perceived biodiversity of urban natural spaces	Perceived value of urban natural spaces	+	[24]
Perceived biodiversity of urban natural spaces	Attractiveness of biodiverse spaces	−	[58]
Perceived biodiversity of urban natural spaces	Perceived neatness of urban natural spaces	−	[58]
Perceived biodiversity of urban natural spaces	Attractiveness of parks for leisure activities	+	[22]
Perceived biodiversity of urban natural spaces	Enjoyability of natural spaces	+	[74]
Perceived level of obstruction	Occurrence of off-trail trampling	+	[46]
Perceived neatness of urban natural spaces	Attractiveness of parks for physical activities	+	[65]
Perceived neatness of urban natural spaces	Support of urban biodiversity conservation	+	[56,59]
Perceived neatness of urban natural spaces	Attractiveness of biodiverse spaces	+	[62]
Perceived neatness of urban natural spaces	Perceived safety of urban natural spaces	+	[74]
Perceived level of obstruction	Occurrence of off-trail trampling	+	[46]
Perceived neatness of urban natural spaces	Attractiveness of parks for physical activities	+	[65]

Table A2. *Cont.*

Cause	Effect	+/−	Reference
Perceived neatness of urban natural spaces	Support of urban biodiversity conservation	+	[59]
Perceived neatness of urban natural spaces	Attractiveness of biodiverse spaces	+	[62]
Perceived neatness of urban natural spaces	Perceived safety of urban natural spaces	+	[67]
Perceived neatness of urban natural spaces	Support of urban biodiversity conservation	+	[56]
Perceived restorative quality of urban natural spaces	Attractiveness of parks for physical activities	+	[65]
Perceived restorative quality of urban natural spaces	Support of urban biodiversity conservation	+	[49]
Perceived restorative quality of urban natural spaces	Enjoyability of natural spaces	+	Hypothesised by authors
Perceived safety of urban natural spaces	Attractiveness of parks for physical activities	+	[65]
Perceived safety of urban natural spaces	Perceived restorative quality of the space	+	[49]
Perceived safety of urban natural spaces	Use of urban natural spaces	+	[42]
Perceived value of urban natural spaces	Use of urban natural spaces	+	[42]
Percentage of area with lawns	Attractiveness of biodiverse spaces	-	[95]
Percentage of area with lawns	Invertebrate species richness	−	[92]
Percentage of area with lawns	Perceived neatness of urban natural spaces	+	[55]
Rate of mowing	Invertebrate species richness	−	[96]
Rate of mowing	Percentage of area with lawns	+	Hypothesised by authors
Rate of weeding	Vegetation levels	−	[21]
Soil erosion	Actual biodiversity of urban natural spaces	+	[25,84]
Structural diversity of vegetation	Invertebrate species richness	+	[97]
Structural diversity of vegetation	Attractiveness of biodiverse spaces	+	[95]
Structural diversity of vegetation	Perceived biodiversity of urban natural spaces	+	[58]
Structural diversity of vegetation	Perceived restorative quality of the space	+	[51]
Structural diversity of vegetation	Avian species richness	+	[85]
Structural diversity of vegetation	Attractiveness of biodiverse spaces	+	[71]
Structural diversity of vegetation	Enjoyability of natural spaces	+	[79]
Support for urban biodiversity conservation	Conservation of urban natural spaces	+	[47]
Support for urban biodiversity conservation	Friendliness of management practices toward biodiversity	+	Hypothesised by authors
Total species richness	Actual biodiversity of urban natural spaces	+	[98]
Total species richness	Perceived restorative quality of the space	+	[64]
Total tree cover	Attractiveness of parks for physical activities	+	[65]
Total tree cover	Degree of canopy closure	+	Hypothesised by authors
Total tree cover	Avian species richness	+	[85,99]
Tree species richness	Avian species richness	+	[100]
Tree species richness	Perceived restorative quality of the space	+	[64]
Tree species richness	Perceived urban natural space biodiversity	+	[64]
Tree species richness	Vegetation species richness	+	Hypothesised by authors
Use of urban natural space	Nature orientation	+	[73]
Use of urban natural space	Nutrient deposition in aquatic areas	+	[26]
Use of urban natural space	Support of urban biodiversity conservation	+	[16]
Use of urban natural space	Occurrence of off-trail trampling	+	[25]

Table A2. *Cont.*

Cause	Effect	+/−	Reference
Use of urban natural space	Nature orientation	+	[17,74,101]
Vegetation levels	Actual biodiversity of urban natural spaces	+	[21,25]
Vegetation levels	Soil erosion	−	[25,102]
Vegetation levels	Density of understory vegetation	+	Hypothesised by authors
Vegetation species richness	Attractiveness of biodiverse spaces	+	[23,77]
Vegetation species richness	Total species richness	+	Hypothesised by authors
Water quality	Actual biodiversity of urban natural spaces	+	[86,103]
Water quality	Attractiveness of biodiverse spaces	+	[79]
Waterlogging	Perceived level of obstruction	+	[46,61]
Wildlife	Perceived restorative quality of the space	+	[7]
Wildlife	Use of urban natural spaces	+	[70]
Wildlife	Enjoyability of natural spaces	+	[69]
Wildlife	Perceived value of urban natural spaces	+	[69]
